# The Effect of Intraoperative Methadone Compared to Morphine on Postsurgical Pain: A Meta-Analysis of Randomized Controlled Trials

**DOI:** 10.1155/2020/6974321

**Published:** 2020-03-27

**Authors:** Mark C. Kendall, Lucas J. Alves, Kristi Pence, Taif Mukhdomi, Daniel Croxford, Gildasio S. De Oliveira

**Affiliations:** Department of Anesthesiology, The Warren Alpert Medical School of Brown University, Providence, RI, USA

## Abstract

**Methods:**

We performed a quantitative systematic review of randomized controlled trials in PubMed, Embase, Cochrane Library, and Google Scholar electronic databases. Meta-analysis was performed using the random effects model, weighted mean differences (WMD), standard deviation, 95% confidence intervals, and sample size. Methodological quality was evaluated using Cochrane Collaboration's tool.

**Results:**

Seven randomized controlled trials evaluating 337 patients across different surgical procedures were included. The aggregated effect of intraoperative methadone on postoperative opioid consumption did not reveal a significant effect, WMD (95% CI) of −0.51 (−1.79 to 0.76), (*P*=0.43) IV morphine equivalents. In contrast, the effect of methadone on postoperative pain demonstrated a significant effect in the postanesthesia care unit, WMD (95% CI) of −1.11 (−1.88 to −0.33), *P*=0.005, and at 24 hours, WMD (95% CI) of −1.35 (−2.03 to −0.67), *P* < 0.001.

**Conclusions:**

The use of intraoperative methadone reduces postoperative pain when compared to morphine. In addition, the beneficial effect of methadone on postoperative pain is not attributable to an increase in postsurgical opioid consumption. Our results suggest that intraoperative methadone may be a viable strategy to reduce acute pain in surgical patients.

## 1. Introduction

Opioid analgesics is still the primary treatment for moderate-to-severe postsurgical pain despite new therapies and interventions [1–3]. Nonetheless, the excessive use of opioids frequently leads to poor postsurgical recovery [4, 5]. In addition, the persistent use of opioids after surgery has been shown to lead to opioid abuse and addiction in selected patients [6–8]. Better use of opioids during surgery is a relevant topic in perioperative medicine.

Commonly used opioids (e.g., morphine) have short duration of action (up to 5 hours) and require frequent dosing to maintain adequate postoperative analgesia [9, 10]. In contrast, methadone has a much longer duration of action (up to 36 hours) that may lead to better postsurgical pain control [11]. Several randomized studies have compared the use of intraoperative methadone to morphine regarding postsurgical analgesia, but they have generated conflicting results. It is currently unknown if the use of intraoperative methadone can lead to better postsurgical pain when compared to intraoperative morphine.

The main objective of the current investigation is to compare the analgesic efficacy of intraoperative methadone to intraoperative morphine for postoperative analgesia outcomes in patients undergoing surgical procedures. We also sought to examine potential side effects related to the use of intraoperative methadone.

## 2. Methods

We carried out a systematic and quantitative meta-analysis following the guidelines of the Preferred Reporting Items for Systematic Reviews and Meta-Analyses statement [12]. The study was registered with the PROSPERO database (CRD42019132112). Institutional review board approval and patient consent were not required. We followed similar methods as previously published by our group [13, 14].

### 2.1. Systematic Search

A comprehensive search of randomized trials investigating intraoperative methadone to morphine on postoperative surgical analgesia was performed using electronic databases PubMed database, Google Scholar, the Cochrane Database of Systematic Reviews, and Embase from inception up to January 2019. The search words “methadone,” “intraoperative methadone,” “postsurgical, “postoperative,” and “pain” were used in various combinations using Boolean operators. Search strategy is shown in Appendix. The search was restricted to adults greater than 18 years of age, and there were no language restrictions. The bibliographies of the identified studies were evaluated and reviewed for additional studies. There was no search performed for unpublished or nonpeer reviewed studies. The initial search identified 382 articles.

### 2.2. Inclusion and Exclusion Criteria

We included single- or double-blinded randomized controlled trials that compared intraoperative methadone with morphine for postoperative analgesia in patients undergoing various surgical procedures. Studies were excluded if a direct comparison of intraoperative methadone and morphine could not be determined. Nonrandomized controlled trials, animal studies, correspondence, or editorials were not considered for inclusion. Included studies reported either on opioid consumption or pain scores at rest as postoperative pain outcomes. No minimum sample size was required for inclusion in the quantitative analysis.

### 2.3. Selection of Included Studies and Data Extraction

Two investigators (MCK and LJA) independently assessed the abstracts and results of the 382 articles obtained from the initial search using the predetermined inclusion and exclusion criteria. The trials that were not relevant based on the inclusion criteria were excluded. Any disagreements encountered during the selection process were resolved by discussion among the evaluators (MCK and LJA). If there was a disagreement among the reviewers, then the final decision was resolved by the senior investigator (GDO). Data extraction was carried out by using a predesigned data collection form. The primary source of data extraction was from either the text or tables. If the data were not found in either location, we extracted the data manually from available figures or plots. The extracted data obtained from studies included sample size, number of participants in treatment groups, type of surgery, methadone dose, morphine dose, postoperative opioid consumption, postoperative pain scores, postoperative nausea and vomiting, and adverse events. Postoperative opioid consumption was converted to intravenous morphine milligram equivalents assuming no cross-tolerance (morEq) [15]. The visual analog scale or numeric rating scale of pain was converted to a 0–10 numeric rating scale (0 = no pain and 10 = extreme pain). Continuous data were recorded using mean and standard deviation. Data outcomes presented as median, interquartile range, or mean ±95% confidence interval (CI) were converted to mean and standard deviation. For studies that did not provide standard deviation, the standard deviation was estimated using the most extreme values. If the same outcome variable was reported more than once, then the most conservative measure was used. Any disagreements were resolved with discussion with the senior author (GDO).

### 2.4. Risk of Bias Assessment

The validity of the included studies was assessed in accordance with Cochrane Collaboration's tool for risk of bias assessment [16]. The tool consists of six domains: selection bias, performance bias, attrition bias, detection bias, reporting bias, and other potential source of bias. Each category was recorded as “low risk,” “high risk,” or “unclear risk” which indicates lack of information or unknown risk of bias. Two investigators (MCK and LJCA) individually assessed the risk of bias of included studies, and any inconsistencies were resolved with discussion with the senior author (GDO).

### 2.5. Primary Outcome

Postoperative opioid consumption (morEq) reported up to 24 hours following surgery.

### 2.6. Secondary Outcomes

Postoperative pain scores (numeric pain rating score, 0 = no pain and 10 = extreme pain) at the postanesthesia care unit (PACU) and 24 hours after surgery, time to first analgesic request (min), and postoperative nausea and vomiting displayed as (*n*) were the secondary outcomes.

### 2.7. Meta-Analyses

The weighted mean differences (WMD) with 95% confidence interval (CI) were calculated and reported for continuous data for total opioid consumption up to 24 hr and pain scores (NRS) at rest up to 24 h. Statistical significance required that the 95% CI for continuous data did not include zero and for dichotomous data, the 95% confidence interval did not include 1.0. Due to the variety of surgical procedures, the random effects model was used in an attempt to generalize our findings to studies not included in our meta-analysis [17]. Asymmetric funnel plots were investigated for publication bias using Egger's regression test [18]. A one-sided *P* < 0.05 was considered as an indication of an asymmetric funnel plot. In the presence of an asymmetric funnel plot, a file drawer analysis was performed, which estimates the lowest number of additional studies that if available would reduce the combined effect to nonsignificance, assuming the average z-value of the combined *P* values of the missing studies to be 0 [19]. Heterogeneity was considered to be high if the *I*^2^ statistic was greater than 50%. If heterogeneity was high, we performed a sensitivity analysis by removing individual studies and examining its effect on the overall heterogeneity. A *P* value < 0.05 was required to reject the null hypothesis. Analyses was performed using Stata version 15 (College Station, Texas) and Comprehensive Meta-analysis software version 3 (Biostat, Englewood, NJ).

## 3. Results

**Figure 1:**
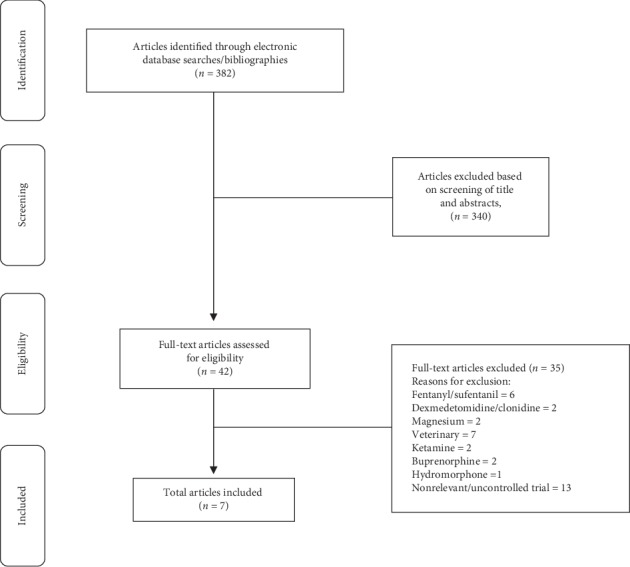
Flow chart of the selection of studies.

Of the 382 articles from our initial search, 340 articles did not meet the inclusion criteria upon further evaluation of the study abstracts. The full text of 42 articles was evaluated, and 35 articles were excluded because they did not meet our inclusion criteria. The specific reasons for exclusions of articles that were fully reviewed are shown in Figure 1. Seven studies met the inclusion criteria, and the characteristics of included trials are listed in Table 1 [20–26]. The evaluated trials included data from 337 subjects and were published between 1986 and 2018. The median and interquartile range (IQR) number of patients in the included studies receiving methadone was 40 (30 to 59). All seven randomized controlled trials were reported on opioid consumption and/or pain scores.

### 3.1. Quality Assessment

**Figure 2:**
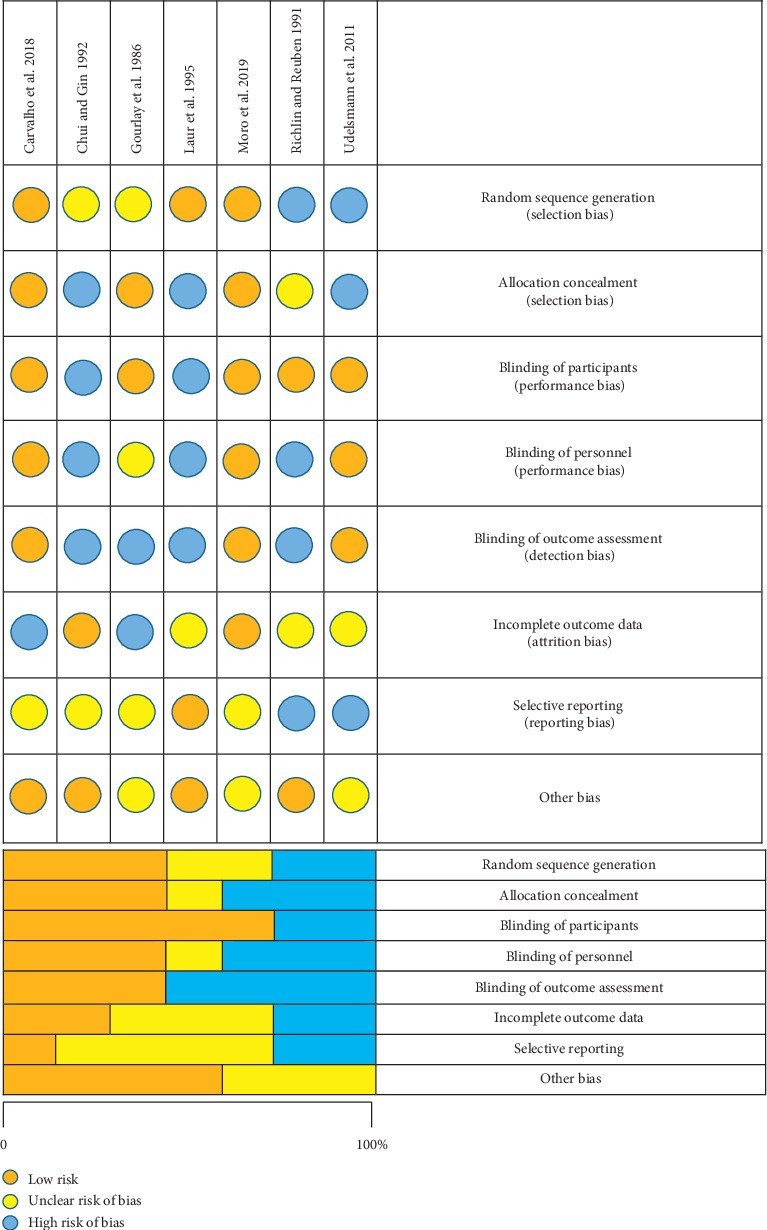
Risk of bias summary and bias graph.

All trials reported inclusion and exclusion criteria and described baseline characteristics. Randomized treatment allocation sequences were created with number generator computer software programs or random number tables in three of the seven studies. Randomized controlled trials describing proper concealment of treatment allocation were described in three trials. A total of three studies described study personnel as blinded to treatment allocation and three studies reported blinding of outcome assessors. The description of patient blinding was clear in five studies. The methodological quality and judgements about each risk of bias domain as a percentage across all included studies are presented in Figure 2.

### 3.2. Postoperative Opioid Consumption Reported up to 24 hours following Surgery

**Figure 3:**
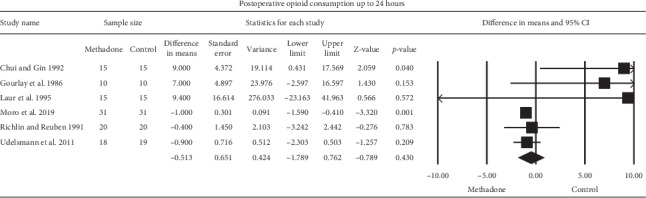
Meta-analysis evaluating the effect of intraoperative methadone on opioid consumption compared to the control at up to 24 hours following surgery. The overall effect of intraoperative methadone versus the control was estimated as a random effect. The point estimate (95% confidence interval) for the overall effect was −0.51 (−1.79 to 0.76), (*P*=0.43) mg IV morphine equivalents. The weighted mean difference for individual studies is represented by the square symbol on the Forrest plot, with 95% CI of the difference shown as a solid line.

The aggregated effect of six studies [21–26] evaluating the effect of intraoperative methadone on postoperative opioid consumption compared to the control up to 24 hours following surgery did not reveal a significant effect in relation to a wide confidence interval, weighted mean difference WMD (95% CI) of −0.51 (−1.79 to 0.76), (*P*=0.43) IV morEq (Figure 3). Heterogeneity was moderate (*I*^2^ = 40%).

### 3.3. Postoperative Pain at PACU after Surgery

**Figure 4:**
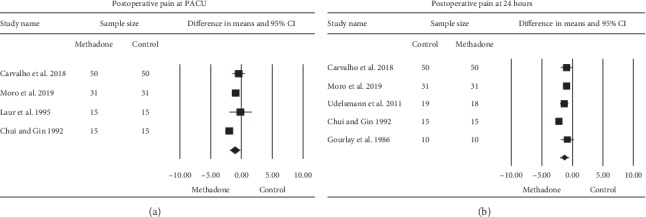
The meta-analysis evaluating the effect of intraoperative methadone on pain scores at PACU (a) and at (b) 24 hours compared to the control was estimated as a random effect. The point estimate (95% confidence interval [CI]) for the overall effect on postoperative pain scores at PACU following surgery was −1.11 (−1.88 to −0.33) (*P*=0.005), (0–10 numerical scale). The point estimate (95% CI) for the overall effect on postoperative pain at 24 hours following surgery was –1.35 (–2.03 to –0.67) (*P* ≤ 0.001), (0–10 numerical scale). The weighted mean difference for individual studies is represented by the square symbol on the Forrest plot, with 95% CI of the difference shown as a solid line.

The effect of the four studies [20–24] evaluating the effect of intraoperative methadone on postsurgical pain compared to the control in PACU following surgery demonstrated a significant effect, WMD (95% CI) of −1.11 (−1.88 to −0.33) (0–10 numerical scale), *P*=0.005 (Figure 4). Heterogeneity was high (*I*^2^ = 81%). Heterogeneity could be partially explained by the type of methadone dose in which the heterogeneity decreased slightly to *I*^2^ = 72% for studies using a high intraoperative methadone dose (>0.25 mg/kg). An examination of the funnel plot did not reveal asymmetry; Egger's regression test revealed to be one-sided, *P*=0.26.

### 3.4. Postoperative Pain at Rest 24 Hours following Surgery

The effect of five studies [20–22, 24, 26] evaluating intraoperative methadone on postoperative surgical pain compared to the control revealed a significant effect WMD (95% CI) of −1.35 (−2.03 to −0.67), (0–10 numerical scale), *P* < 0.001, (Figure 4). Heterogeneity was high (*I*^2^ = 89%). Heterogeneity could be partially explained by the type of methadone dose in which the heterogeneity slightly decreased to *I*^2^ = 81% for studies using a high methadone dose (>0.25 mg/kg). An examination of the funnel plot did not reveal asymmetry; Egger's regression test revealed to be one-sided, *P*=0.37.

### 3.5. Time to First Analgesic Request in the Postoperative Period

Five studies [20, 22, 23, 25, 26] evaluated the effect of intraoperative methadone compared to the control on the time to first analgesic dose in the postoperative period which did not demonstrate an effect, WMD (95% CI) of 378.86 (−6.65 to 764.38) (*P*=0.054). Heterogeneity was high (*I*^2^ = 88%). Heterogeneity could be partially explained by the type of surgery in which the heterogeneity decreased to *I*^2^ = 71% for noncardiac surgeries.

### 3.6. Postoperative Nausea and Vomiting

**Figure 5:**
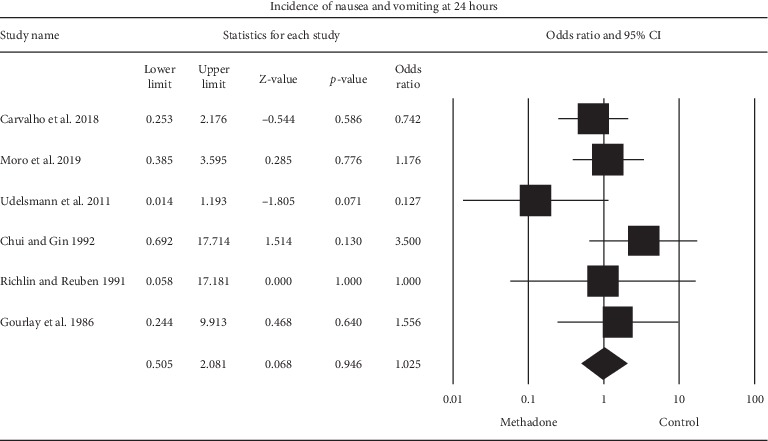
Random effects meta-analysis evaluating the effect of intraoperative methadone on nausea and vomiting compared to the control. Squares to the right of the middle vertical line indicate that intraoperative methadone was associated with increased odds of nausea, whereas squares to the left of the middle vertical line show that intraoperative methadone was associated with decreased odds of nausea. The horizontal lines represent the 95% CI, and the diamond shape represents the overall effect of intraoperative methadone on postoperative nausea and vomiting compared to the control. CI = confidence interval.

In the six studies [20–22, 24–26] that reported on nausea and vomiting, the aggregated effect of the studies that investigated intraoperative methadone on postoperative nausea and vomiting compared to the control did not reveal a significant effect, OR (95% CI) of 1.025 (0.51 to 2.08) (*P*=0.95) (Figure 5). Heterogeneity was low, *I*^2^ = 18%.

### 3.7. Adverse Events

Four studies reported no adverse events (respiratory depression and excessive sedation) or did not report any events. One study reported that patients who received intraoperative methadone experienced more sedation compared to the control group at 24 hours after surgery [25]. In contrast, Moro et al. reported that patients allocated to the intraoperative morphine group experienced more sedation (Ramsey score ≥4) compared to those in the methadone group during the postoperative period [24]. No cardiac disturbances were reported in the included studies.

## 4. Discussion

The most important finding of the current investigation was the reduction of postoperative pain in patients who received intraoperative methadone compared to intraoperative morphine across multiple surgical procedures. Patients in the methadone group reported less pain in the immediate postoperative phase (e.g., PACU). In addition, patients also reported a reduction of pain at 24 hours after surgery. Taken together, our results suggest that intraoperative methadone is an efficacious strategy to reduce postsurgical pain.

Our results are clinically important as pain remains to be poorly controlled after surgery [27–30]. Adequate postsurgical pain control has been correlated with improved patient satisfaction [31, 32]. Therefore, it is possible that using intraoperative methadone in favor of morphine as part of a multimodal analgesia regimen may affect patients' postoperative recovery process and improve patient satisfaction after surgery.

Another important finding of our current investigation was the lack of opioid sparing effects of intraoperative methadone when compared to morphine. This excludes a possible increase in opioid consumption which is the explanation for lower postoperative pain observed in the methadone group. In addition, it may also explain the lack of effect of methadone on reducing opioid-induced side effects (e.g., postoperative nausea and vomiting).

Methadone not only activates the same opioid receptors as morphine, but also blocks the N-methyl-D-aspartate (NMDA) receptor reducing hyperalgesia, enhancing analgesia, and weakening the development to tolerance [33]. A single dose of methadone has a rapid effect, but the maximum effect can often be achieved after several days of use [34]. This suggests that methadone may be a good strategy for treatment of postoperative pain lasting for days. In fact, a prior randomized study has confirmed the opioid sparing effect of methadone used postoperatively as a patient-controlled analgesic strategy in patients undergoing total hip arthroplasty when compared to morphine [35].

Cardiac disturbances, such as QT interval prolongation, have been reported in 20% of individuals taking methadone, particularly in patients receiving high doses of methadone for a long period of time ranging from 40 mg/day to 700 mg/day [36]. The absence of reporting of cardiac disturbances in the present study may be due to short duration of use and overall low total methadone consumption. Future studies examining the effect of methadone on heart rhythm abnormalities after surgery are warranted.

The findings of our study should only be interpreted within the context of its limitations. First, in order to minimize clinical heterogeneity, we compared methadone to morphine only. It is possible that the use of intraoperative methadone can have greater postoperative analgesic effects when compared to other commonly used intraoperative opioids (e.g., fentanyl) [37, 38]. Secondly, we limited our comparison to the intraoperative phase. It is conceivable that methadone may have greater analgesic effects when used for prolonged periods of time. Lastly, we included a large number of surgical procedures in an attempt to improve the generalizability of our findings, but this has resulted in significant heterogeneity. Nonetheless, we used the random effect model for all the analyses and were able to explain some of the heterogeneity based on the type of surgical procedure.

In summary, our study shows that the use of intraoperative methadone reduces postoperative pain when compared to morphine. In addition, the beneficial effect of methadone on postoperative pain does not appear to be attributable to an increase in postsurgical opioid consumption. Side effects related to the use of intraoperative methadone were not distinguishable between the ones observed with intraoperative morphine. Our results suggest that intraoperative methadone may provide some benefit to mitigate postoperative pain in surgical patients. Future studies with larger sample sizes and longer follow-up periods with more comprehensive reporting are warranted to draw more robust conclusions.

## Figures and Tables

**Table 1:** Summary of study characteristics included in analysis.

Authors	Year of publication	Procedures	Number treatment/control	Treatment	Administration time	Method of extraction
Carvalho et al [20]	2018	Coronary artery bypass grafting	31/31	0.1 mg/kg methadone 0.1 mg/kg morphine	Induction	Text Table
Chui and Gin [21]	1992	Abdominal Hysterectomies	15/15	0.25 mg/kg methadone 0.25 mg/kg morphine	Induction	Text Table
Gourlay et al [22]	1986	Cholecystectomy Vagotomy Nissen fundoplication	10/10	0.3 mg/kg morphine 0.3 mg/kg methadone	10 min after induction	Text Table
Laur et al [23]	1995	Orthopedic surgery	15/15	0.3 mg/kg methadone 0.3 mg/kg morphine	25% patient positioning; 50% induction; 25% before incision	Text Table
Moro et al [24]	2019	Laparoscopic Cholecystectomy	50/50	0.1 mg/kg methadone 0.1 mg/kg morphine	End of anesthesia	Text Table
Richlin and Reuben [25]	1991	Lower abdominal surgery	20/20	0.3 mg/kg morphine 0.3 mg/kg methadone	Right after induction	Text Table
Udelsmann et al [26]	2011	Cardiac surgery with extracorporeal circulation	18/19/18	0.3 mg/kg methadone 0.3 mg/kg morphine 2 mL normal saline	Induction	Table Text

## Appendix

### A. Search Strategy, PubMed

(((“methadone”[MeSH Terms] OR “methadone”[All Fields]) AND (“surgery”[Subheading] OR “surgery”[All Fields] OR “surgical procedures, operative”[MeSH Terms] OR (“surgical”[All Fields] AND “procedures”[All Fields] AND “operative”[All Fields]) OR “operative surgical procedures”[All Fields] OR “surgery”[All Fields] OR “general surgery”[MeSH Terms] OR (“general”[All Fields] AND “surgery”[All Fields]) OR “general surgery”[All Fields])) AND Randomized Controlled Trial[ptyp] AND “humans”[MeSH Terms] AND “adult”[MeSH Terms]) AND (Randomized Controlled Trial[ptyp] AND “humans”[MeSH Terms] AND “adult”[MeSH Terms])

### B. Cochrane Library

“methadone” in Title Abstract Keyword OR intraoperative methadone in Title Abstract Keyword OR postsurgical pain in Title Abstract Keyword AND pain in Title Abstract Keyword.

### C. Embase

methadone”/exp OR methadone OR “intraoperative methadone” OR “postsurgical pain”:au AND “randomized controlled trial (topic)”/de AND “randomized controlled trial (topic)”/de AND [adult]/lim
